# 1006 The Presentation of Microstomia in Burn Survivors

**DOI:** 10.1093/jbcr/iraf019.537

**Published:** 2025-04-01

**Authors:** Miranda Yelvington, Bernadette Nedelec, Samuel Mandell, Haig Yenikomshian, Jeffrey Schneider

**Affiliations:** Arkansas Children’s Hospital; McGill University; University of Texas Southwestern / Parkland; University of California Keck School of Medicine; Spaulding Rehabilitation Hospital, Harvard Medical School

## Abstract

**Introduction:**

Microstomia, or small oral aperture, often results from deep facial burns. This condition develops as scars form around the perioral region, narrowing the oral opening. It can impact oral hygiene and a person’s ability to eat, and often produces undesirable cosmetic outcomes. While perioral contractures can be prevented and mitigated, it remains unclear which injury factors cause the highest risk. This study examines the prevalence of microstomia and its relationship to demographic and injury-related factors.

**Methods:**

Data from a multicenter longitudinal database from 2001-2005, were analyzed. The frequency of microstomia (yes/no) was examined at discharge. Summary statistics were used to describe clinical characteristics and burn location in pediatric and adult burn survivors to assess for predictors of microstomia. Wilcoxon Rank Sum and Fisher’s Exact tests were used to test for significant differences between those with and without microstomia.

**Results:**

There were 91 pediatric and 214 adult burn survivors during our time period. Among pediatrics, 11% (10) presented with microstomia. Among adults, 4.7% (10) presented with microstomia. In both pediatric and adult survivors, those with microstomia had significantly larger TBSA burned (p=0.002 pediatric, p=0.033 adult), higher rate of inhalation injury (p=0.002 pediatric only), more days on ventilation (p=0.003, p< 0.001), higher rate of head, neck, or face graft (p=0.012 p=0.005), higher rate of nasolabial contractures (p< 0.001, p< 0.001), and higher rate of neck contractures (p=0.033, p< 0.001). No differences were found in age, sex, race, ethnicity, etiology of burn, or discharge location.

**Conclusions:**

The results of this analysis suggest that burn survivors with larger burn size and greater length of time requiring ventilation are more likely to develop of microstomia than those with small burns and less ventilator time. Other risk factors include the requirement for head, neck or face graft, which suggest an association with deeper burns.

**Applicability of Research to Practice:**

This study highlights the risk factors for the development of microstomia. Knowledge of these risk factors indicates that therapists should prioritize early microstomia prevention, even during times of ventilation, especially in those with larger burn injuries. The high incidence of associated nasolabial and neck contractures in those with microstomia highlights the importance of consideration of cutaneokinematics and skin recruitment and suggests that perioral areas, as well as surrounding regions, should be considered together when developing treatment and prevention programs. Special attention should be given to burn survivors with larger burns and those requiring prolonged ventilation, as they are at greater risk for microstomia.

**Funding for the Study:**

NIDILRR #90DPBU0008, 90DPGE0004. Partial Support was obtained from Grant #79136-BOS-23 and 79138-BOS-23.

